# Methylation of tumor suppressor genes in ovarian cancer

**DOI:** 10.3892/etm.2012.715

**Published:** 2012-09-18

**Authors:** FILIZ OZDEMIR, JULIDE ALTINISIK, ATES KARATEKE, HAKAN COKSUER, NUR BUYRU

**Affiliations:** 1 Department of Medical Biology, Istanbul University, Cerrahpasa Medical Faculty;; 2 Department of Gynecology and Obstetrics, Zeynep Kamil Maternity and Child Research and Training Hospital, Istanbul, Turkey

**Keywords:** ovarian cancer, methylation, methylation-specific multiplex ligation-dependent probe amplification, tumor suppressor gene

## Abstract

Aberrant methylation of gene promoter regions is one of the mechanisms for inactivation of tumor suppressor genes in human malignancies. In this study, the methylation pattern of 24 tumor suppressor genes was analyzed in 75 samples of ovarian cancer using the methylation-specific multiplex ligation-dependent probe amplification (MS-MLPA) assay. Of the 24 tumor suppressor genes examined, aberrant methylation was observed in 17. The three most frequently methylated genes were CDKN2B, CDH13 and RASSF1, followed by ESR1 and MLH1. Methylation frequencies ranged from 1.3% for CDKN2A, RARβ, CASP8, VHL and TP73 to 24% for CDKN2B. The corresponding normal DNA from each patient was also investigated. Methylation was detected in tumors, although not in normal tissues, with the exception of two samples, indicating aberrant methylation in tumors. Clear cell carcinoma samples exhibited a higher frequency of CDKN2B promoter hypermethylation compared to those of other histological types (P=0.05). Our data indicate that methylation of the CDKN2B gene is a frequent event in ovarian carcinogenesis and that analysis of only three genes is sufficient to detect the presence of methylation in 35% of ovarian cancer cases. However, more studies using a much larger sample size are needed to define the potential role of DNA methylation as a marker for ovarian cancer.

## Introduction

Ovarian cancer is the most lethal tumor of the female genital tract and the second most frequent gynecological cancer (1). Approximately 1.5% of females develop ovarian cancer in their lifetime. As ovarian cancer has few symptoms in its course, most cases are diagnosed in the late stages. If diagnosed during the early stages, most cases are curable. The overall response rate is 80% in advanced cases. However, the 5-year survival rate is only 15–20%, mainly due to chemoresistance. Current prognostic indicators using clinicopathological variables, including stage and grade (2), neither accurately predict clinical outcome nor provide biological insight into the disease. Thus, a better understanding of the molecular mechanisms responsible for ovarian cancer development and progression is likely to aid the improvement of the diagnosis and treatment of the disease.

Similar to other tumor types, ovarian cancer is thought to arise following the activation of oncogenes and inactivation of tumor suppressor genes. In addition to genetic alterations, epigenetic abnormalities, such as changes in genomic DNA methylation patterns, are associated with all human malignancies (3–5). Changes in the DNA methylation pattern in cancer include global hypomethylation of the CpG dinucleotides in repetitive DNA regions (6) in conjunction with hypermethylation of CpG island promoter-associated genes (3,7). It has been increasingly shown over the past 10 years that the CpG islands in the promoter regions of a large number of genes, which are mostly unmethylated in normal tissues, are methylated to varying degrees in human cancer (4,5,7). Since aberrant gene methylation is one of the earliest molecular alterations occurring during cancer (8–10), it has emerged as a promising strategy for the early detection of cancer (11). In ovarian cancer, a growing number of genes have been recognized as undergoing aberrant methylation at CpG islands, suggesting this to be an important molecular mechanism in the development of ovarian carcinoma (12). In ovarian cancer, hypermethylation has been found to be associated with the inactivation of virtually all pathways involved in cancer development, including DNA repair, cell cycle regulation, apoptosis, cell adherence and detoxification pathways (13–20). However, most of these studies have focused on a single candidate gene and the reported frequencies and disease specificities vary between independent studies. These discrepancies most likely reflect differences in the populations that were studied and the methods used. Previous studies have suggested that methylation profiles of cancers are tumor type- and ethnicity-specific (21,22).

Therefore, in the present study, we investigated aberrant methylation of promoter regions in 24 methylation-prone tumor suppressor genes in ovarian cancer tumor samples. The association between the methylation status of these genes and major clinicopathological parameters of patients was also evaluated.

## Materials and methods

### Tissue samples and DNA extraction

In this study, tumor and adjacent normal tissue samples obtained from 75 ovarian cancer patients at the time of resection were used. Genomic DNA was extracted from the tissue samples of patients using the High Pure PCR template preparation kit (Roche Diagnostics, Mannheim, Germany) according to the manufacturer’s instructions. This study was approved by Ethics Committee of Istanbul University, Cerrahpasa Medical Faculty (Istanbul, Turkey) and all patients provided written informed consent.

### Methylation-specific multiplex ligation-dependent probe amplification (MS-MLPA)

Gene methylation status was evaluated by MS-MLPA using the ME001B tumor suppressor kit (MRC-Holland, Amsterdam, The Netherlands). In this type of assay, the probes added to the samples are amplified, not sample nucleic acids. Amplification of probes by polymerase chain reaction (PCR) depends on the presence of probe-target sequences in the sample. Each probe consists of two oligonucleotides that hybridize to adjacent sites of the target sequence. The probes contain a recognition sequence for the methylation-sensitive restriction enzyme, *Hha*I. Upon digestion of the sample DNA probe hybrids with *Hha*I, unmethylated probe-recognition sequences in the sample do not generate a signal. By contrast, the target region is successfully amplified if the site is methylated. The ME001 tumor-suppressor kit contains 26 probe sequences corresponding to a set of 24 tumor suppressor genes frequently silenced by methylation and 15 control probes that are not affected by methylation-sensitive *Hha*I restriction digestion.

### Methylation determination

Following amplification, the products were analyzed by sequence type capillary electrophoresis (ABI 310; Applied Biosystems, Foster City, CA, USA). Subsequent quantification of the methylation status was performed by comparing the relative signal peaks from the digested and undigested samples. Data analysis was performed by exporting the peak areas to the excel-based Coffalyser analysis program (MRC-Holland) and the normalized areas from the digested and undigested samples were compared in order to determine the methylation status of the individual genes. Methylation was scored as positive when the calculated methylation levels were >25%. Any methylation rate below this level was regarded as background, depending on the results of negative controls and a mathematical algorithm reported previously (23).

### Methylation-specific PCR (MS-PCR)

The MLPA results were further confirmed by MS-PCR. Since the RASSF1 gene was one of the genes revealing the highest frequency of positivity, this gene was selected for further analysis by MS-PCR. The DNA samples were converted by sodium bisulphite using the EZ DNA Methylation Kit (Zymo Research, Irvine, CA, USA) following the manufacturer’s instructions. The modified DNA was amplified using methylated- and unmethylated-specific primers to amplify the same fragment within the promoter of RASSF1. The primers were UMF: 5′-TTTGGTTGGAGTGTGTTAATGTG-3′ and UMR: 5′-CAAACCCCACAAACTAAAAACAA-3′ for the unmethylated and MF: 5′-GTGTTAACGCGTTGCGTATC-3′ and MR: 5′-AACCCCGCGAACTAAAAACGA-3′ for the methylated sequences. PCR was performed in a total volume of 50 *μ*l containing 100 ng of bisulfite-converted DNA by an initial denaturation for 10 min at 95°C, followed by 35 cycles of denaturation at 95°C for 1 min, annealing at 60°C for 1 min and extension at 72°C for 1 min. The products were analyzed on 2% agarose gels.

## Results

### Methylation frequencies

In this study, promoter regions from a total of 24 tumor suppressor genes were analyzed for methylation of CpG islands in the tumors and adjacent normal tissues of 75 ovarian cancer cases. In 17 genes, methylation was detected in at least one tumor sample. Methylation of the ATM, HIC1, CDKN1B, PTEN and FHIT genes was not detected in any tumor sample, whereas low frequencies were observed for the DAPK1 (3 samples), CHFR, IGSF4, GSTP1 (2 samples), CDKN2A, RARβ, CASP8, VHL and TP73 (1 sample) genes. Methylation frequencies ranged from 1.3% for TP73, VHL, CASP8, RARβ and CDKN2A to 24% for CDKN2B. The most frequently methylated genes were CDKN2B (24% of cases), CDH13 (16% of cases) and RASSF1 (12% of cases). The results are shown in Table I. Overall, 30 of 75 (40%) tumor DNA samples showed methylation at least in one gene. None of the tumor samples were methylated at >7 sites. Six of the 24 genes (TIMP3, CHFR, IGSF4, GSTP1, CASP8 and VHL), not previously reported to be methylated in ovarian cancer, were methylated in our study group at a low frequency (1.3-5.3%). Two DNA samples from non-tumor tissues also showed methylation of the same genes which were methylated in the corresponding tumor tissues. This may be due to the contamination of these samples from adjacent malignant cells.

### Correlation between clinicopathological characteristics and methylation

Although the initial population comprised 75 patients, reliable clinicopathological data were available only for 38 patients. Of these tumors, 34 were epithelial and 4 were non-epithelial. None of the non-epithelial tumor samples showed methylation in any of the 24 tumor suppressor genes. Hypermethylation was found in six of 24 (25%) papillary serous, 3 of 6 mucinous (50%) and 2 of 2 clear cell (100%) carcinoma tumors. The remaining 2 epithelial samples also did not show methylation of any gene (Table II). To confirm the MS-MLPA results, the methylation status of the RASSF1 gene was analyzed by MS-PCR by conventional bisulfite treatment and using a commercial DNA modification kit. In all specimens, the experiments revealed identical results by MS-MLPA and MS-PCR. The clinical stage was determined according to the International Federation of Gynecology and Obstetrics (FIGO) criteria and histological subtypes were evaluated according to the World Health Organization (WHO) classification.

P-values were determined using Fisher’s exact test, P<0.05 was considered to indicate a statistically significant result. Statistical analyses of the associations between the pathological characteristics of the patients and methylation were performed for the three most frequently methylated genes. There was no association between the methylation status of the genes and tumor stage or age of the patients. Methylation of the CDKN2B gene was more frequent in clear cell carcinoma than other histological types (P=0.049).

## Discussion

Methylation is the main epigenetic event in humans, and changes in the methylation pattern play an important role in tumorigenesis (5,24,25). Early diagnosis is critical for the successful treatment of numerous types of cancer, including ovarian cancer. As aberrant methylation is frequently observed in cancer development and is thought to be one of the earliest molecular changes in carcinogenesis (26), the detection of alterations in DNA methylation patterns has potential application to the detection of early-stage or potentially premalignant disease. A specific pattern of CpG island hypermethylation existing in each type of human cancer was first reported for colorectal cancer by Costello *et al* (21) and was later confirmed by Esteller *et al* (27). For ovarian cancer, the methylation of different genes has been investigated in cell lines or in a small number of tumors. However, methylation of single genes may have limited value in clinical applications. Therefore, analysis of the methylation status of multiple genes simultaneously may provide a more sensitive and specific assay for the molecular classification and prognosis of ovarian cancer. The MS-MLPA assay is a new and sensitive method of detecting aberrant methylation in multiple genes in a single reaction (23). Therefore, in the present study the MS-MLPA method was applied to the simultaneous analysis of the methylation status of distinct tumor suppressor genes in ovarian cancer.

A large number of genes have been identified as hypermethylated and silenced in ovarian cancer, with the reported frequency of methylation varying widely between independent studies (12). Therefore the clinical relevance of these observations remains uncertain. In 2001, Strathdee *et al* (19) reported that primary ovarian cancer may exhibit multiple methylator phenotypes, including known tumor suppressor genes. In previous studies, the most frequenty methylated genes were reported as the OPCML, Hsulf-1, GATA4, DAPK1 and CDH13 genes (12,15,16,28–30). In agreement with these reports, the most frequently methylated genes in our study were the CDKN2B, CDH13 and RASSF1 genes. CDKN2B is a cell cycle control gene and several lines of evidence indicate that variants of these genes, particularly CDKN2A, are crucial in ovarian cancer (31–33). Results of a recent study have indicated that the underexpression of certain cell cycle regulatory proteins, such as CDKN2A, is a predictive marker for shorter overall survival in ovarian carcinoma (34). In most cases, loss of the protein occurs due to the deletion of the gene (35). Yang *et al* also investigated methylation in gynecological cancers and reported that BRCA1, p14, p16 and PTEN are differentially silenced by hypermethylation (36). However, certain investigators have also suggested that CDKN2B does not have an important role in ovarian carcinogenesis, due to the absence of mutations and homozygous deletion of the gene in ovarian cancer (37). In contrast to CDKN2A, the methylation and mRNA expression of CDKN2B in ovarian cancer have not been extensively investigated. Aberrant methylation in the promoter region of the gene has been found in a number of types of cancer, including hematological malignancies (38–40) and lung (41) and breast cancer (42). The reported rates of CDKN2B methylation in ovarian cancer tissue ranges from 0 to 30.8% (18,37,43). One study indicated that methylation of CDKN2B was present in 30.8% (43) of ovarian cancer cases and was associated with disease progression, whereas other studies showed that methylation of CDKN2B is a rare event (18,37). The discrepancies among these results may be due to the differences in methods for methylation detection, inclusion of different histological types or the differences in the ethnic origins of the patients. However, in our study, CDKN2B was the most frequently methylated gene among 24 tumor suppressor genes. Our results are comparable to those reported by Kudoh *et al* (32) and Liu *et al* (43). However, we found a statistically borderline correlation between CDKN2B methylation and clear cell carcinoma. Clear cell tumors are generally regarded as the most aggressive histological type of epithelial tumors. In our study, all the clear cell carcinoma samples showed methylation of the CDKN2B gene promoter. Our results indicate that the CDKN2B gene is silenced in ovarian cancer, and especially in clear cell carcinoma, by promoter methylation instead of deletion or mutation. However, the number of patients with clear cell carcinoma was low (2.6%) in our study group.

CDH13 was the second most frequently methylated gene in our study group. CDH13 (H-cadherin) is a cell adherence protein and the loss of CDH13 expression in malignant ovarian tumors has been reported previously (44). Although hypermethylation of CDH13 is common in breast and lung cancer (45), there are only a few reports in the literature investigating CDH13 methylation in ovarian cancer (15,16,44,46). According to these studies, CDH13 has been found to be methylated in 13–67% of ovarian cancer tissue samples. Although it was the second most frequently methylated gene in our study group, the methylation frequency was not as high as previously reported (46). This difference may reflect population differences, since epigenetic alterations may be different in different ethnic groups. Our results support the role of the CDH13 gene in ovarian cancer.

The third most frequently methylated gene was RASSF1. RASSF1 is a tumor suppressor gene, the inactivation of which is suggested in the development of different types of human cancer (47). Although it may be inactivated by deletion or point mutations, the most common contributor to loss or reduction of RASSF1 function is transcriptional silencing of the gene by inappropriate promoter methylation. Previous studies have indicated that RASSF1 is frequently methylated in breast, lung and ovarian cancers (42,48,49). Although there are conflicting reports in the literature with regard to the methylation of the RASSF1 gene in ovarian tumors, our results are in agreement with the findings of Agathanggelou *et al* (49), which showed that RASSF1 is methylated in 10% of ovarian cancer cases, irrespective of grade. In this study, no statistically significant association was found between RASSF1 hypermethylation and clinicopathological characteristics, such as age, tumor grade and the histological tumor type.

We observed a lower degree of hypermethylation (1.3–8%) in the promoters of 14 genes, which act in different cellular pathways. These results indicate that in ovarian cancer, several genes which have a role in different pathways may be silenced by promoter hypermethylation.

## Figures and Tables

**Table I:** Methylation frequencies of the tumor suppressor genes in ovarian cancer.

Tumor suppressor gene	Chromosomal position	Methylated tumors, n=75 (%)
CDKN2B	9p.21	18 (24)
CDH13	16q24.2	12 (16)
RASSF1	3p21.3	9 (12)
ESR1	6q25.1	6 (8)
MLH1	3p21.3	6 (8)
TIMP3	22q12.3	4 (5.3)
APC	5q21	4 (5.3)
BRCA1	17q21	4 (5.3)
DAPK1	9q34.1	3 (4)
CHFR	12q24.33	2 (2.6)
IGSF4	11q23	2 (2.6)
GSTP1	11q13	2 (2.6)
CDKN2A	9p21	1 (1.3)
RARβ	3p24	1 (1.3)
CASP8	2q33-q34	1 (1.3)
VHL	3p26-p25	1 (1.3)
TP73	1p36	1 (1.3)
ATM	11q22.3	-
HIC1	17p13.3	-
CDKN1B	12p13.1	-
PTEN	10q23.31	-
BRCA2	13q12.3	-
CD44	11p13	-
FIHT	3p14.2	-

**Table II:** Association between gene promoter methylation and clinicopathological characteristics of ovarian cancer.

	CDKN2B methylation		CDH13 methylation		RASSF1 methylation		ESR1 methylation	
Clinicopathological parameters	+	-	+	-	+	-	+	-
Age (years)^a^								
≤50	1	13	0	14	0	14	0	14
>50	7	16	3	20	0	23	4	19
Histological type								
Epithelial ovarian tumor								
Serous	4	20	2	22	0	24	1	23
Mucinous	2	4	1	5	0	6	1	5
Clear cell	2	0	0	2	0	2	2	0
P-value	0.0499							
Endometriosis	0	1	0	1	0	1	0	1
Brenner	0	1	0	1	0	1	0	1
Non-epithelial ovarian tumor								
Granulosa cell	0	4	0	4	0	4	0	4
Grade								
I	1	3	0	4	0	4	1	3
II	2	8	2	8	0	10	1	9
II	5	19	1	23	0	24	2	22

a Clinicopathological data were available for 38 patients, however, information on the age of one of these patients was missing.
